# Fabrication of Ultra-Thin Printed Organic TFT CMOS Logic Circuits Optimized for Low-Voltage Wearable Sensor Applications

**DOI:** 10.1038/srep25714

**Published:** 2016-05-09

**Authors:** Yasunori Takeda, Kazuma Hayasaka, Rei Shiwaku, Koji Yokosawa, Takeo Shiba, Masashi Mamada, Daisuke Kumaki, Kenjiro Fukuda, Shizuo Tokito

**Affiliations:** 1 Research Center for Organic Electronics (ROEL), Graduate School of Science and Engineering, Yamagata University, 4-3-16 Jonan, Yonezawa, Yamagata 992-8510, Japan; 2 Innovation Center for Organic Electronics (INOEL), Graduate School of Science and Engineering, Yamagata University, 1-808-48, Arcadia, Yonezawa, Yamagata 992-0119, Japan; 3 Japan Science and Technology Agency, PRESTO, 4-1-8, Honcho, Kawaguchi, Saitama, 332-0012, Japan

## Abstract

Ultrathin electronic circuits that can be manufactured by using conventional printing technologies are key elements necessary to realize wearable health sensors and next-generation flexible electronic devices. Due to their low level of power consumption, complementary (CMOS) circuits using both types of semiconductors can be easily employed in wireless devices. Here, we describe ultrathin CMOS logic circuits, for which not only the source/drain electrodes but also the semiconductor layers were printed. Both p-type and n-type organic thin film transistor devices were employed in a D-flip flop circuit in the newly developed stacked structure and exhibited excellent electrical characteristics, including good carrier mobilities of 0.34 and 0.21 cm^2^ V^−1^ sec^−1^, and threshold voltages of nearly 0 V with low operating voltages. These printed organic CMOS D-flip flop circuits exhibit operating frequencies of 75 Hz and demonstrate great potential for flexible and printed electronics technology, particularly for wearable sensor applications with wireless connectivity.

In recent years, there has been great interest in the use of organic thin film transistor (OTFT) device technology in next-generation thin film electronics, due to the performance enhancements enabled by its use in applications such as flexible displays^1^,^2^, large-area sensors^3^,^4^, radio frequency identification (RFID) tags^5^,^6^, and ultrathin electronics^7^,^8^,^9^. In particular, ultrathin devices have received significant consideration for use in practical wearable devices and RFID tags that operate wirelessly and should consume low power and have a small circuit footprint. A complementary (CMOS) logic configuration is suitable for such devices because of its low power consumption and smaller physical layout compared with the unipolar p-MOS or n-MOS circuit configurations.

Fabrication of OTFT-based circuits using conventional printing technologies is considered a promising approach due to advantages such as low capital investment, efficient utilization of material, and low production cost, motivating extensive research on the fabrication of organic CMOS integrated circuits either entirely or in part by using printing methods. Some studies have examined the printing of circuits that use organic semiconductors (p and n type)^10^,^11^,^12^, while others have investigated hybrid configurations using both inorganic and organic semiconductors^13^,^14^. However, these reports used low performance n-type organic semiconductors or vacuum deposited electrodes.

Currently, the optimization of the electrical characteristics of p-type and n-type OTFT devices requires the modification of the electrode surfaces by using a self-assembled monolayer (SAM)^15^, oxide layer^16^, or polymer layer^17^ processes for the different semiconductor materials. Unfortunately, complex processes such as SAM patterning using a plasma treatment^5^ are required to form the source and drain electrodes of each of n and p type OTFT devices on the same substrate, which are unsuitable for volume production and prevent the use of organic transistors in practical applications. To overcome this limitation, we have devised a structure for forming vertically stacked p-type and n-type OTFT devices. By combining printing methods with this stacked device structure and using an ultrathin substrate, the processing time is short and total device thickness remains very thin. There have been some reports regarding the use of stacked structures to obtain high density integrated circuits^18^, to control of OTFT device characteristics^19^, and to combine inorganic and organic device processes^20^ for the fabrication of the integrated circuits. However, an electrode surface modification technology for the solution-processed semiconductors has not yet been developed.

In this study, we have demonstrated a CMOS inverter logic gate as well as ring oscillator and D-flip flop (FF) circuits on a micron-thick substrate by stacking the solution-processed OTFT devices. To reduce the complexity of the CMOS inverter circuit fabrication, we developed a sophisticated stacked-structure printing process. This fabrication process exhibits the following advantages: 1) simple electrode surface modification with different SAM layers, 2) use of a common gate electrode layer to simplify the circuit layout, 3) top-gate, bottom-contact (TG-BC) device structure to maximize and take advantage of the performance of the n-type OTFT. As a result, excellent electrical characteristics were achieved in both p and n type OTFT devices with printed electrodes and solution-processed semiconductor layers. In addition, the fabricated CMOS inverter circuit operated successfully at low operating voltages of 1–10 V, as did the master–slave (MS)-type D-FF circuit with the output buffer consisting of forty (40) transistors on the ultrathin substrate. Furthermore, we successfully fabricated these integrated circuits on a 1-μ m thick film substrate and have demonstrated their ability to operate even when this film is compressed.

## Results

### Fabrication of stacked organic complementary logic circuits on ultrathin substrates

**Figure 1:**
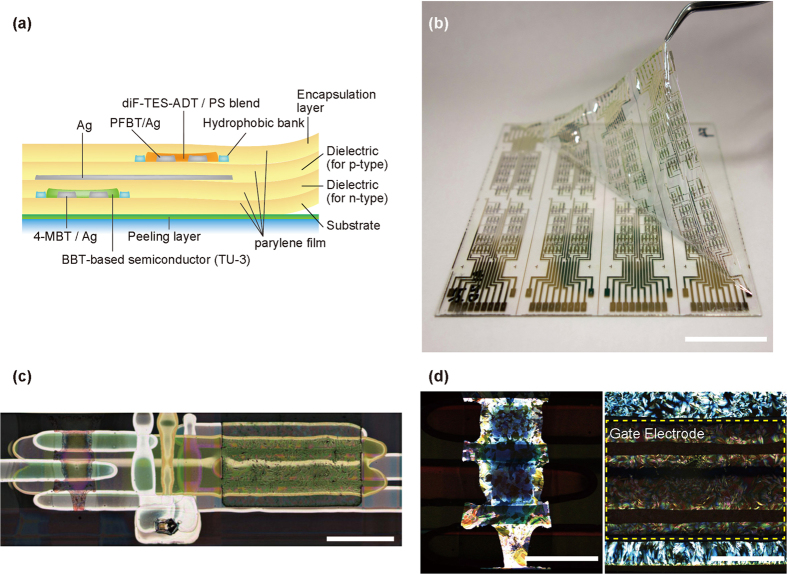
Ultrathin organic CMOS logic circuits using a stacked structure. (**a**) Complete structure for the stacked devices. (**b**) Photograph of organic CMOS logic circuit on a one-micron (μ m) substrate. The total thickness was less than 3 μ m. Scale bar, 25 mm. (**c**) Image of the CMOS inverter constituting the D-FF circuit. Scale bar, 500 μ m. (**d**) Polarized microscope image of the diF-TES-ADT/PS blend and TU-3. Scale bar, 250 μ m.

The fabricated inverter logic gate has a stacked configuration consisting of n-type OTFT with TG-BC structure overlapping on a p-type OTFT with BG-BC structure of (Fig. 1a). This device structure has several advantages, including, and most importantly, the fact that the electrode surface modification process in the complementary configuration is simplified. In an organic transistor with printed electrodes, surface treatment of the source and drain electrodes using a SAM layer is very important for improving the OTFT characteristics^22^. In this device configuration, the fabrication processes for the p-type and n-type OTFT devices are performed separately. Therefore, the electrode surface treatment for both OTFT device types can be carried out using a simple immersion process. This is in contrast to surface treatment methods using an O_2_ plasma previously reported in work by Battiato *et al.*^5^, which is necessitated by the formation of both OTFT device types on the same layer and is one of the inevitable drawbacks that reduces the benefits of the printing processes. In another study, a staggered device structure was reported to be electrically superior to a planar structure^23^. Therefore, we used a TG-BC structure for the n-type OTFT device.

Figure 1b shows a photograph of the ultrathin organic CMOS logic circuit. The procedure for device fabrication on an ultrathin film substrate was reported previously^21^. The substrate consists of parylene diX-SR is only 1 μ m thick, such that the total thickness of the stacked OTFT devices is less than 3 μ m. The fabricated devices can be easily peeled from the supporting glass plate after completing the entire fabrication process. All device layers were fabricated with printing processes except for deposition of the parylene layer. These ultrathin devices demonstrate the feasibility of the multi-layer stacked device structures for highly integrated circuits with lightweight that can adhere to any surface and over large areas.

Figure 1c shows an optical microscope image of the fabricated inverter circuit. To obtain geometric information, we observed the p-type OTFT and n-type OTFT devices with a polarization microscope after fabricating the two OTFT devices in separate areas. Figure 1d shows the polarization microscope image of p-type and n-type semiconductors. Both semiconductor films are polycrystalline, which we concluded is the likely reason for the reduced variations in n-type OTFT device electrical characteristics. (see Supplementary Information, Fig. S1).

### Electrical performance of p- and n-type OTFT devices in CMOS inverters

**Figure 2:**
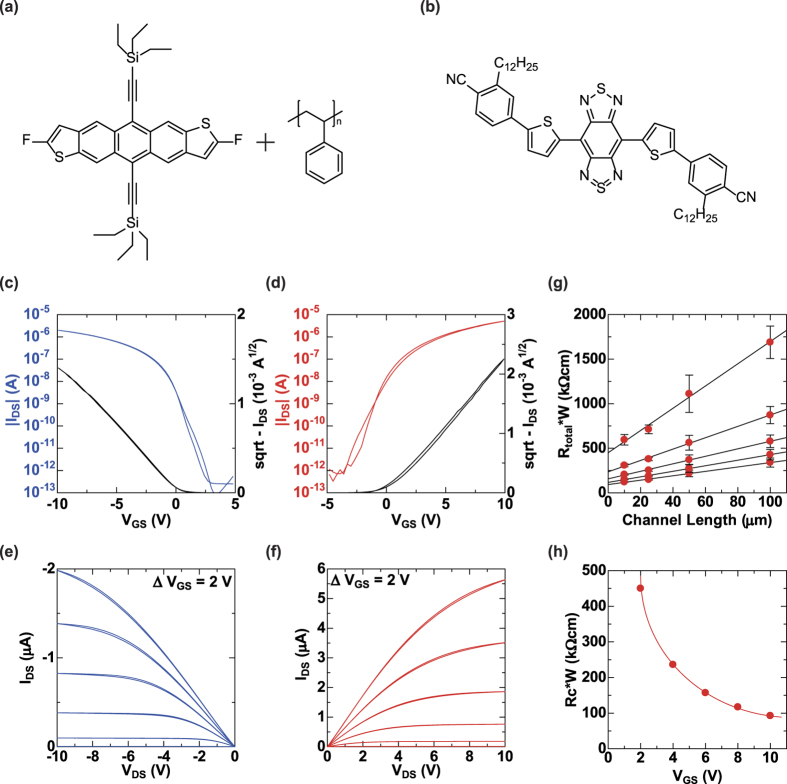
Molecular structure and transistor features of printed OTFTs. Molecular structure of (**a**) the p-type semiconductor (diF-TES-ADT and PS) and (**b**) the n-type semiconductor (TU-3). Transfer characteristics of (**c**) the p-type OTFT with *V*_*DS*_ =  − 10 V and (**d**) the n-type OTFT with *V*_*DS*_ =  10 V. Saturation mobilities are 0.34 and 0.21 cm^2^ V^−1^ sec^−1^, respectively. Output characteristics of the (**e**) p-type OTFT and (**f**) n-type OTFT. (**g**) Channel width-normalized total resistance (R_total_) as a function of channel length. (**h**) Width-normalized contact resistance (R_c_) as a function of *V*_*GS*_. The width-normalized contact resistance was 92.6 k Ω cm at *V*_*GS*_ =  10 V.

Figure 2a,b show the chemical formula of the organic semiconductors used in the complementary (CMOS) inverter gate. For the p-type semiconductor, we used an ink consisting of a diF-TES-ADT and PS blend, which is known to be a high performance organic semiconductor^24^,^25^,^26^,^27^. A novel solution-processable n-type semiconductor called TU-3^28^,^29^,^30^ was developed in collaboration with Ube Industries, Ltd. Although its basic chemical structure is rigid benzobis(thiadiazole) (BBT), TU-3 shows high solubility in many common organic solvents.

We evaluated the effectiveness of the SAM modification process on a printed silver electrode using photoemission spectroscopy. The work function of the bare electrodes was 4.8 eV, changing to 5.3 and 4.0 eV after pentafluorobenzenethiol (PFBT) and 4-methylbenzenethiol (4-MBT) treatments, respectively. (see Supplementary Information, Fig. S2)

We then measured the transistor characteristics of the OTFT devices that make up the inverter circuit in ambient air conditions. Figure 2c,d show the transfer characteristics of the p-type and n-type OTFT devices, respectively, and Fig. 2e,f show the corresponding output characteristics. The channel length (L) and width (W) of the fabricated p-type and n-type OTFT devices were L/W =  52/612 and 41/2012 μ m, respectively. These OTFT devices exhibited excellent TFT device electrical performance at relatively low operating voltages of 10 V. For the p-type OTFT, the estimated hole-mobility and threshold voltage were 0.34 cm^2^ V^−1^ sec^−1^ and 0.17 V, respectively, and the on/off current ratio was greater than 10^7^. For the n-type OTFT, the estimated electron-mobility and threshold voltage were 0.21 cm^2^ V^−1^ sec^−1^ and 0.53 V, and the on/off current ratio was greater than 10^7^. These threshold voltage values were very close to the ideal value of 0 V.

Accordingly, we have successfully achieved n-type OTFT device performance comparable to that of the p-type OTFT device for balanced complementary logic functionality. These good output characteristics suggest that the contact resistance for each TFT device type is low. Since the contact resistance (R_c_) of OTFT with printed electrode and semiconductor layers provides important information for understanding the electrical transport, there have been some reports on the R_c_ of p-type OTFT devices^15^,^16^,^21^. However, no R_c_ values have been reported for n-type OTFT devices. We estimated R_c_ for the n-type transistors using the transmission line method (TLM)^31^. Here, R_c_ is given by


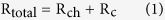


where R_total_ is the resistance between the source and drain electrodes and the R_ch_ is the channel resistance. To precisely calculate the contact resistance using equation (1), we fabricated TFT devices with various channel lengths and their total resistance values estimated from the output characteristics. Figure 2g,h show the TLM plots used to calculate the R_c_ values in n-type OTFTs. R_c_ is estimated to be as low as 92.6 k Ω cm at *V*_*GS*_ =  10 *V*. Thus, a low R_c_ for the n-type TFT comparable to that of the p-type TFT was achieved by modifying the electrode surface with a SAM layer for the n-type semiconductor layer and a high-performance printed CMOS OTFT circuit was realized.

### Electrical performance of CMOS inverters

To investigate the feasibility of using the stacked structure for the organic CMOS circuits, we have evaluated the inverter logic gate circuit, which is the most basic component for the complementary integrated circuits. The inverter gate operates as follows: when the input voltage (*V*_*in*_) is low, the output voltage (*V*_*out*_) is high; on the contrary, when Vin is high, *V*_*out*_ becomes low. Ideally, *V*_*out*_ should be equal to *V*_*DD*_ at a high input voltage.

**Figure 3:**
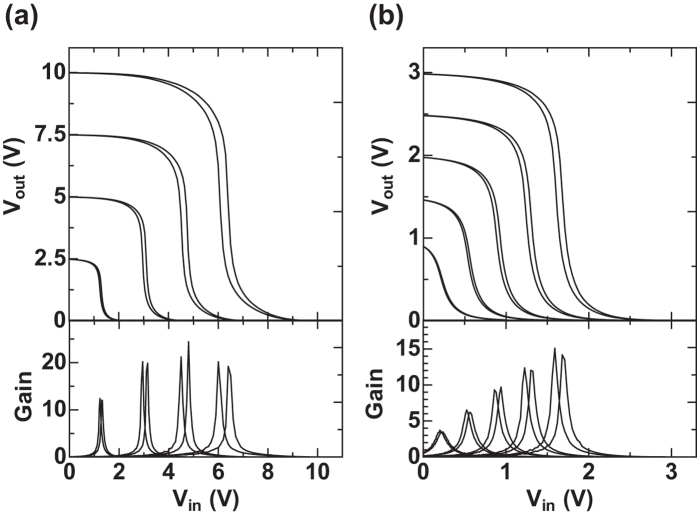
Characteristics of complementary inverter circuits. Static transfer characteristics of the inverter and signal gain as a function of input voltage (Vin) with (**a**) operating voltages 2.5–10 V and (**b**) 1–3 V. Inset figure shows a complementary inverter gate circuit.

The inverter gate circuit requires the combination of p-type and n-type OTFT devices as shown in the inset of Fig. 3a. Generally, the static and dynamic electrical performances of lf inverter gate circuits are evaluated. In this section, we describe the voltage gain characteristics, trip point and noise margin of the static characteristics with dynamic characteristics described in the subsequent section. Figure 3a,b display the voltage transfer characteristics at various *V*_*DD*_ supply voltages. The sharp switching is one of the major advantages of the CMOS logic gates. Inversion characteristics with small hysteresis as low as 3% of *V*_*DD*_ are obtained for *V*_*DD*_ ranging from 1 to 10 V. Furthermore, voltage gain values are as high as ~20 at a *V*_*DD*_ of 5–10 V, ~10 at a *V*_*DD*_ of 2.5–3 V, and ~3 at a *V*_*DD*_ of 1 V. Here, the voltage gain is defined as the absolute value of ∆ *V*_*out*_/∆ *V*_*in*_. The trip point is defined as the input voltage *V*_*in*_ at *V*_*out*_ =  Vin. In an ideal inverter gate, the trip point should be equal to one half of *V*_*DD*_. Figure 3a,b show that the trip points are indeed close to half of the *V*_*DD*_ value and are 6.3, 4.7, 3.1 and 1.3 V at *V*_*DD*_ of 10, 7.5, 5 and 2.5 V, respectively.

The noise margin is defined as the side of the largest square that can be inscribed between the transfer characteristics. The maximum noise margin is equal to half of the supply voltage. Our inverter circuit shows a noise margin of greater than 50% at a *V*_*DD*_ from 2 to 10 V. This is the highest noise margin reported so far for printed CMOS organic inverters^32^.

### D flip-flop based on CMOS inverters

To further demonstrate the capability of our process, we have fabricated a practical D-FF circuit is a basic circuit to configure registers and bit counters.

**Figure 4:**
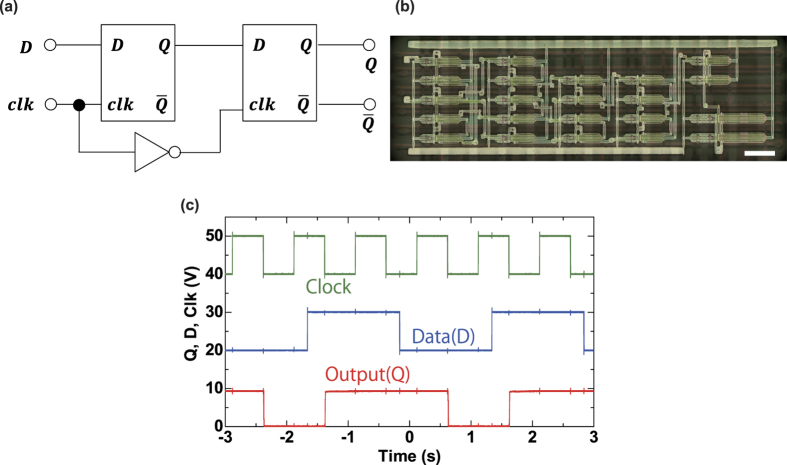
Characteristics of the D-FF circuit. (**a**) Block diagram of the MS-type D-FF circuit. (**b**) Optical microscope image of fabricated D-FF circuit including 40 TFT devices. Scale bar, 2 mm. (**c**) Dynamic characteristics of the D-FF circuits with a clock frequency of 1 Hz and an operating voltage of 10 V.

A complementary MS-type D-FF circuit construction is depicted in Fig. 4a. This circuit is composed of forty (40) OTFT devices including a buffer inverter (more detailed circuits diagrams are provided in the supplementary information Fig. S3). Figure 4b shows the images of the fabricated devices. Even though some reports show a simpler D-FF circuit configurations, such as a transmission gate type and edge-trigger type^5^,^33^,^34^,^35^,^36^,^37^, we have evaluated a conventional MS-type consisting forty (40) OTFT devices to validate the high yield of our stacked structure. Figure 4c shows the input-output characteristics of the fabricated D-FF circuits at a supply voltage of 10 V. All electrical measurements for the D-FF circuits were carried out in the atmosphere.

This MS-type D-FF circuit reads the state of the input at a clock state of high and changes the output state at the falling edge of the clock. When the clock state is high, the output state is held. At a falling clock edge, the output state is changed or held to coincide with the input D-state. For example, the low output state is changed to and the high output state is held with the same state as the high input state at a falling clock edge. The timing chart in Fig. 4c shows that the fabricated circuit operates properly.

We then changed the clock frequency to evaluate the operating speed of the circuits. We applied a square-wave clock signal with a duty cycle of 50% and showed that the D-FF functions correctly at a clock frequency of 75 Hz (13.3 ms/clock cycle). This value is approximately equal to the 80 Hz estimated by the circuit simulation (see Supplementary Information Fig. S4). In previously reported work by Schwartz *et al.*^38^, an MS-type FF with printed electrodes was operated at a speed of 10 ms/clock cycle and a supply voltage of 20 V. Therefore, a clock speed of 13.3 ms is a compatible value with the supply voltage of 10 V.

To demonstrate the light weight and flexibility of the circuit, we operated the circuit while under the influence of strong current of air. Even under these conditions, the circuit functioned without any degradation in electrical performance (see Supplementary Information movie S1).

### Mechanical compressibility of the integrated circuits

**Figure 5:**
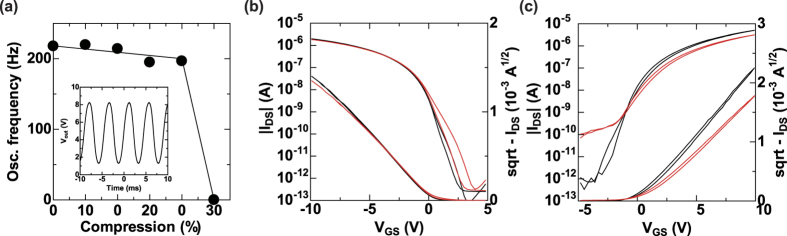
Effects of compression on the complementary ring oscillator and OTFT devices. (**a**) Changes in oscillation frequency as a function of compressive strain. Inset figure shows output characteristic of a ring oscillator with an operating voltage of 10 V and an oscillation frequency of 217.7 Hz. Transfer characteristics of (**b**) p-type and (**c**) n-type OTFTs operated under no strain (black line) and 50% compressive strain (red line), demonstrating the mechanical stability of the stacked structure.

The stability of the fabricated circuits under compression was evaluated by measuring the oscillation frequency of a 3-stage ring oscillator. Following the procedure used in our previous work^21^, we affixed the circuit’s film substrate to a pre-stretched elastomer. When the elastomer was released from the stretched condition to the original condition, the circuit film was compressed. Figure 5a shows the oscillation frequency of the ring oscillator at the corresponding compression ratios. In the original state, for which the compression ratio is defined as 0%, the oscillation frequency was 217.7 Hz and the delay time per stage was 0.77 ms. Such a delay time is considered excellent for a solution-processed ring oscillator with low operation voltages^39^. Upon compression of the film by 20%, the oscillation frequency changed slightly to 194.9 Hz and the delay per stage was 0.86 ms. However, the devices could not function at a compression ratio of 30%. To investigate the origin of this effect, we evaluated the transistor properties in a compressed state. Figure 5a,b show the transfer characteristics of the p-type and n-type OTFT devices at conditions before and after 50% compression, respectively. The mobility of p-type TFT decreased by 6.2% after the compression, and the threshold voltage shifted by − 0.11 V.

Compared to the p-type OTFT device, the performance of the n-type OTFT device was much worse after the compression. The mobility was reduced by 40%, and the threshold voltage was shifted by − 0.42 V.

In the organic semiconductor, there are decrease of injection efficiency due to the increase of potential barrier at the electrode interface and increase of the contact resistance which induce the mobility degradation and threshold voltage shift simultaneously^40^. Therefore, it is suggested that the potential-barrier increased by compression is one of the cause for these characteristics variations.

Because normal transistor characteristics were obtained even when the film was compressed by 50%, we suspect that the ring oscillator stopped oscillating because of poor contact through a via hole to the power-supply interconnect lines. Since this is not an essential process for the OTFT device and circuit, this malfunction of the circuit can be eliminated by improving the printing processes for the interconnect and the integrity of the via hole.

## Discussion

In conclusion, we have successfully fabricated complementary (CMOS) logic circuits with low-voltage operation using OTFT devices with a novel stacked configuration, which employs a SAM modification treatment using a simple immersion process and uses printing methods to form the source/drain electrode and semiconductor layers on ultrathin film substrates, thus realizing all-printed n-type and p-type OTFT devices with a total thickness of 3 μ m or less.

Both OTFT devices showed excellent carrier mobilities of more than 0.2 cm^2^ V^−1^ sec^−1^, and the carrier mobility of the n-type semiconductor was the highest ever reported for printed complementary circuits. The threshold voltage of the fabricated devices was sufficiently low; hence, the digital logic gate was driven at a low operating voltage of only 5 V. In addition, the fabricated inverter gate was able to perform the proper inversion operations at very low operating voltages of 1.5 V and was shown to operate successfully even under compression.

As a result, we are the first to demonstrate printed complementary CMOS OTFT process and circuits on an ultrathin film substrate with a thickness of 1 μ m. We believe that this technology will be instrumental in the development of future flexible and printed electronic applications, particularly wearable sensors.

## Methods

### Semiconductor

Mesitylene-based 2,8-difluoro-5,11-bis(triethylsilylethynyl)anthradithiophene (diF-TES-ADT)^41^ 2 wt%/Polystyrene (PS, average Mw ~280,000, Sigma-Aldrich) 0.5 wt% blend solution was used for the p-type semiconductor^24^,^25^,^26^,^27^. The novel n-type semiconductor based on benzobis (thiadiazole) (BBT) derivative (TU-3, Ube Industries, Ltd.)^28^,^29^,^30^ was prepared in a 0.06 wt% solution for printing using 1-methylnaphthalene (TCI).

### Device fabrication

The complementary (CMOS) inverter logic gates have a stacked structure, and the device structure for each of the n-type and p-type OTFT devices are top-gate, bottom-contact (TG-BC) and bottom-gate, bottom contact (BG-BC), respectively.

The CMOS logic gates are based on solution-processable semiconductors and printed electrodes that were fabricated on a 1-μ m-thick parylene film substrate^21^. A 700-μ m-thick glass substrate with a peeling layer was used as a supporting plate to enable easier handling of the 1-μ m- thick film.

A 1 wt% amorphous fluoropolymer (Teflon AF 1600, DuPont) in Fluorinert (FC-43, 3M Co.) was used for the peeling layer, and was formed by spin-coating.

A 1-μ m-thick parylene layer (diX-SR, KISCO LTD.) was deposited on the peeling layer with a PDS 2010 LABCOTER 2 parylene coater (Specialty Coating Systems) and then the substrate was annealed at 120 °C for 1 hour in the ambient conditions.

We have prepared devices using the NPS-JL (Harima Chemicals Inc.) which is commonly available silver nanoparticle ink containing 52–57 wt% silver nanoparticles with an average diameter of 7 nm in tetradecane, resistivity 9.5 μ  Ω cm, viscosity 8–15 mPa s^42^. This silver nanoparticles ink was printed with a piezoelectric ink-jet printer (DMP-2831, Fujifilm Dimatix Co.) using a 10 pL cartridge for all electrodes, including the source, drain, and gate electrodes and the interconnect lines. The cartridge temperature and the plate temperature were maintained at 40 °C and 50 °C, respectively. The drop spacing (DS) was 60 μ m; this value was optimized to obtain fine printed electrodes. After the printing processes, the substrates were sintered at 120 °C for 30 min in ambient conditions. Next, solution amorphous fluoropolymer was patterned using a dispenser system (IMAGEMASTER 350, Musashi Engineering, Inc.) to create hydrophobic bank layers that confined the deposited n-type organic semiconductor layer, and were subsequently cured at 100 °C for 30 min in air. Prior to the deposition of the semiconductor layer, the source and drain electrode surfaces were modified by immersing them in a 10 mM solution of 4-MBTin isopropanol for 5 min, and then rinsing with pure isopropanol^43^. The n-type semiconductor solution (TU-3) was applied in the areas defined by the bank layers using the dispenser system. During the dispenser printing process, the substrate and nozzle temperatures were 60 °C and 30 °C, respectively. Dispensing conditions were optimized to improve the performance of the n-type OTFT, whereby the discharge pressure was 10 kPa, and the discharge time was about 200 ms. The conditions for the crystal growth process (drying process of 1-methylnaphthalene) were maintained in atmosphere at 60 °C for 30 min. After drying the n-type semiconductor layer, the substrates were annealed at 120 °C for 30 min. in a nitrogen ambient. The parylene layer was deposited and annealed under the same conditions as 1 μ m substrate layers to form the 260-nm-thick gate dielectric layer of the n-type OTFT. The silver nanoparticle ink was printed and sintered under the same conditions as the source and drain electrodes to form gate electrodes for both the n-type and p-type OTFT devices at the same time. After printing the gate electrodes, the parylene layer was deposited under the same conditions as that for the n-type OTFT device dielectric layer to form the gate dielectric layer for the p-type OTFT device. The via holes were formed by a YAG laser (VL-C30, V-Technology Co., Ltd.), and the wavelength output 355 nm. Next, the silver nanoparticle ink was patterned and sintered in the same conditions as the other electrodes to form the source and drain electrodes for the p-type OTFT device. Next, hydrophobic bank layers were printed by using the dispenser system for defining the channel width of the p-type OTFT devices under the same conditions as for the n-type OTFT device layer. Prior to the printing of the p-type semiconductor layer, the source and drain electrode surfaces were modified by immersing them in a 30 mM solution of PFBT in isopropanol for 5 min, and then rinsing with pure isopropanol^44^,^45^. Finally, a mesitylene-based solution containing a p-type semiconductor was printed in the areas defined by bank layer using the ink-jet printer. During this time, the cartridge temperature and the plate temperature were maintained at 30 °C. After drying the semiconductor layer, a 540 nm-thick parylene layer was deposited as the encapsulation layer. These solution-processable CMOS inverter gate fabrication processes were used to create the ring oscillator and D-FF fcircuits.

### Device characterization

The electrical characteristics of the fabricated organic TFT devices and static performance of logic circuits were measured using a semiconductor parameter analyzer (Keithley, model 4200-SCS), whereby all electrical measurements were carried out in air. Photographs of the fabricated devices were obtained using a laser microscope (Olympus, model OLS-4000). The metal surface work function was measured using photoemission spectroscopy (Riken Keiki, model AC-3). The dynamic performance of the integrated logic circuits was measured by using a function generator (Keysight, model 33522B) and an oscilloscope (Tektronix, model DPO2024B) with a passive voltage probe (Tektronix, model TPP0200).

## Additional Information

**How to cite this article**: Takeda, Y. *et al.* Fabrication of Ultra-Thin Printed Organic TFT CMOS Logic Circuits Optimized for Low-Voltage Wearable Sensor Applications. *Sci. Rep.* **6**, 25714; doi: 10.1038/srep25714 (2016).

## Supplementary Material

Supplementary Information

Supplementary Movie
