# Supplementary material for: Fabrication of Ultra-Thin Printed Organic TFT CMOS Logic Circuits Optimized for Low-Voltage Wearable Sensor Applications
Source: Sci Rep. 2016 May 9;6:25714. doi: 10.1038/srep25714 (PMC4860580; doi:10.1038/srep25714)
Supplement: Supplementary Information [file srep25714-s1.doc]

**Supplementary Information**

Fabrication of Ultra-Thin Printed Organic TFT CMOS Logic Circuits Optimized for Low-Voltage Wearable Sensor Applications

Yasunori Takeda^1^, Kazuma Hayasaka^1^, Rei Shiwaku^1^, Koji Yokosawa^1^, Takeo Shiba^1^, Masashi Mamada^2^, Daisuke Kumaki^1^, Kenjiro Fukuda^1,3^, Shizuo Tokito^1,a^

^1^Research Center for Organic Electronics (ROEL), Graduate School of Science and Engineering, Yamagata University, 4-3-16 Jonan, Yonezawa, Yamagata 992-8510, Japan

^2^Innovation Center for Organic Electronics (INOEL), Graduate School of Science and Engineering, Yamagata University, 1-808-48, Arcadia, Yonezawa, Yamagata 992-0119, Japan

^3^Japan Science and Technology Agency, PRESTO, 4-1-8, Honcho, Kawaguchi, Saitama, 332-0012, Japan

^a^ Author to whom correspondence should be addressed: [tokito@yz.yamagata-u.ac.jp](mailto:tokito@yz.yamagata-u.ac.jp)


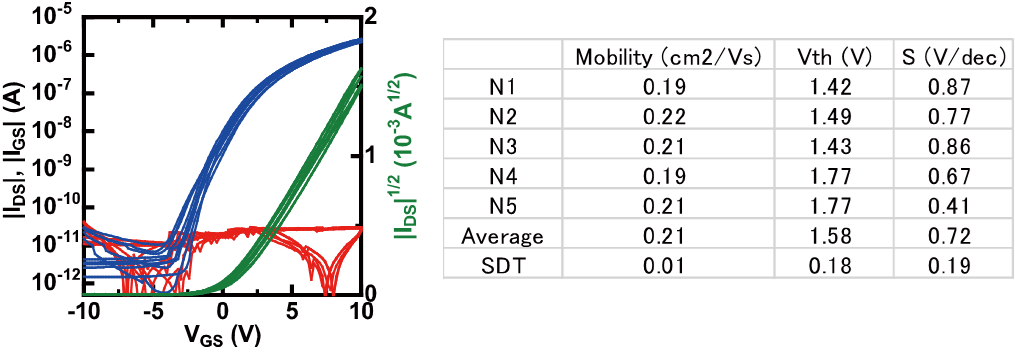


**Supplementary Figure S1 | Variation in electrical characteristics of the n-type OTFT.**

Transfer characteristics of fabricated for five (5) n-type OTFT devices. The variation in characteristics was very small, and the standard deviation of the mobility was only 0.01 cm^2^ V^-1^ s^-1^. The table summarizes the electrical performance of the n-type OTFT as measured for all the devices.


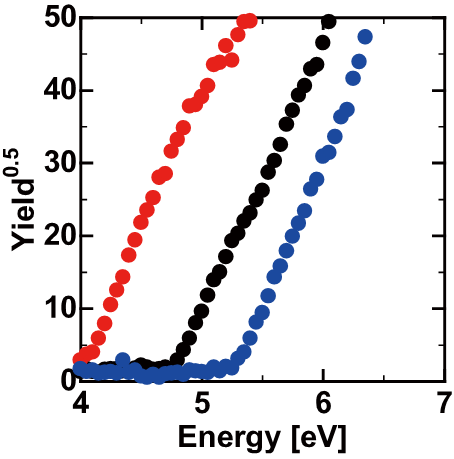


**Supplementary Figure S2 | Photoemission spectroscopy result of the SAM-treated ink-jet printed Ag electrode.**

Work function of the bare electrode (black plots) and that of the electrode after PFBT (blue plots) and 4-MBT (red plots) treatment were 4.8, 5.3 and 4.0 eV, respectively.


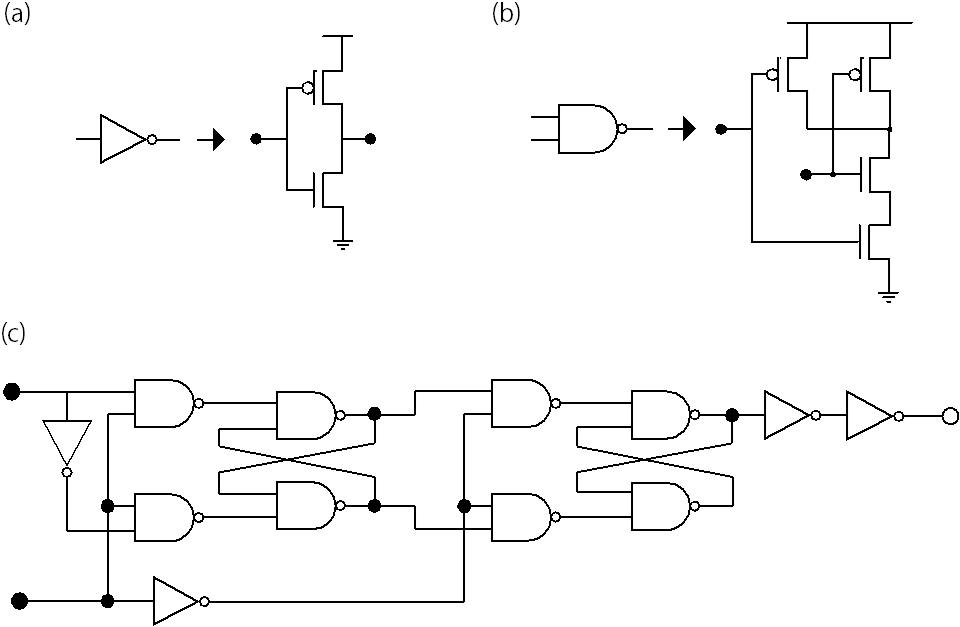


**Supplementary Figure S3 | MIL symbol diagrams of CMOS circuits.**

MIL symbol diagram and circuit diagram of (**a**) a CMOS inverter and (**b**) a NAND gate. (**c**) MIL symbol diagram of MS-type D-FF circuits with two buffers.


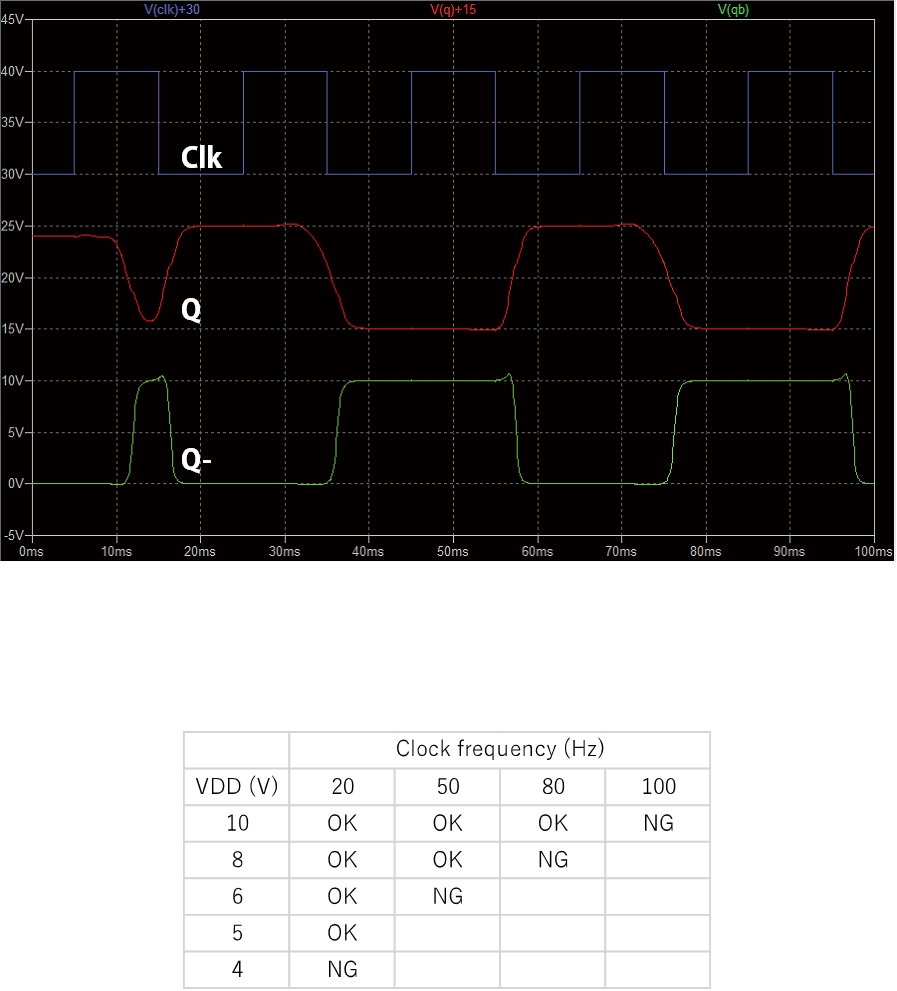


**Supplementary Figure S4 | Simulation results of D-flip flop circuits with a divider mode.**

Simulation result of the clock divided at a clock frequency of 50 Hz. The divider mode results of each clock frequency and each operation voltage are presented. Since large-scale integrated circuits such as the those in RFID tags and microprocessors are designed using a complementary OTFT configuration, OTFT modeling using the circuit simulator “SPICE” is essential. We developed a SPICE model for OTFT device to consider the of TFT performance parameters for their operation in integrated circuits, and successfully fabricated high-performance OTFT circuits.

**Supplementary Movie S1 | Demonstration of D-FF circuit operation.**

Demonstration of D-FF circuit operation in the peeled-off condition. The D-FF circuit was operated without any degradation in performance under the influence of a strong current of air. The measured operating voltage was 10 V and clock frequency was 1 Hz.
